# Subtotal Epiglottectomy and Ablation of Unilateral Arytenoid Cartilage as Surgical Treatments for Grade III Laryngeal Collapse in Dogs

**DOI:** 10.3390/ani12091118

**Published:** 2022-04-27

**Authors:** Francesco Collivignarelli, Amanda Bianchi, Massimo Vignoli, Andrea Paolini, Ilaria Falerno, Giulia Dolce, Paolo Cortelli Panini, Roberto Tamburro

**Affiliations:** 1 Faculty of Veterinary Medicine, University of Teramo, 64100 Teramo, Italy; fcollivignarelli@unite.it (F.C.); apaolini@unite.it (A.P.); ifalerno@unite.it (I.F.); rtamburro@unite.it (R.T.); 2 Roma Sud Veterinary Clinic, Via Pilade Mazza, 00173 Roma, Italy; gveteunipg@gmail.com (G.D.); p.cortellipanini@gmail.com (P.C.P.)

**Keywords:** subtotal epiglottectomy, ablation of unilateral arytenoid cartilage, stage III laryngeal collapse, brachycephalic upper airway obstruction

## Abstract

**Simple Summary:**

Laryngeal collapse is a condition characterized by the medial deviation of the cuneiform cartilage caused by upper airway obstruction. The condition is graded in three stages: stage III is defined as the collapse of the corniculate processes of the arytenoids and the destructuration of the dorsal portion of the rima glottidis. The surgical treatments for this condition include unilateral arytenoid laryngoplasty with cricoarytenoid lateralization combined with thyroarytenoid caudo-lateralization or permanent tracheostomy. Subtotal epiglottectomy was initially described for dogs with epiglottic retroversion. In the present study, subtotal epiglottectomy in association with the ablation of unilateral arytenoid cartilage is proposed as an alternative treatment for 16 dogs with stage III laryngeal collapse. The procedure aims to increase the air passage through the larynx, decrease negative intraglottic pressure, and reduce airway resistance.

**Abstract:**

Stage III laryngeal collapse is defined as the collapse of the corniculate processes of the arytenoid cartilages and the destructuration of the dorsal portion of the rima glottidis. The primary cause is chronic upper airway obstruction, and the condition is often present in brachycephalic dogs. The treatment is still controversial; the patients are generally treated with a permanent tracheostomy. This article reports the authors’ experience with 16 dogs affected by stage III laryngeal collapse treated with subtotal epiglottectomy and the ablation of unilateral arytenoid cartilage. Before the surgery, all of the dogs underwent an effort test to classify the clinical severity of the disease and an endoscopic examination of the airways to determine the stage of severity of the laryngeal collapse. One month after surgery, the effort test was repeated in order to evaluate the clinical outcome. One year after surgery, the owners of 12 patients rated their dogs as follows: excellent in five cases, good in five cases, and fair in two cases. According to this pilot study, epiglottectomy associated with the photoablation of unilateral arytenoid cartilage increases airway flow, and thus may be considered a valid surgical procedure to treat dogs affected by grade III laryngeal collapse.

## 1. Introduction

Laryngeal collapse is defined as a medial deviation of the cuneiform cartilage caused by chronic intraluminal airway pressures that lead to upper airway obstruction, and is commonly observed in dogs with brachycephalic obstructive airway syndrome [1]. Laryngeal tract disorders in both brachycephalic and non-brachycephalic dogs are believed to be a consequence of turbulent airflow caused by chronic upper airway obstruction and high negative pressure that is established in the pharynx [2].

In 1960, Leonard graded laryngeal collapse in three stages: stage I is the reversion of the laryngeal saccules, stage II is the lack of tension in the cuneiform processes of the arytenoid cartilage and its medial displacement, and stage III is the collapse of the corniculate processes of the arytenoid cartilages and the loss of the dorsal arch of the rima glottidis [3,4].

Arytenoid lateralization and permanent tracheostomy were the most common surgical procedures described to treat stage III laryngeal collapse [5,6,7,8]. Unilateral arytenoid laryngoplasty, which consists of cricoarytenoid lateralization combined with thyroarytenoid caudo-lateralization, allowed us to obtain good long-term outcomes in brachycephalic dogs affected by laryngeal collapse [9]. Moreover, the authors observed that the untreated arytenoid cartilage may continue to collapse medially post-operation as a consequence of progressive airway obstruction [8]. Permanent tracheostomy is another described treatment in dogs with laryngeal collapse, but this option is often considered unacceptable by the owners due to stoma management or severe complications that require surgical revision or medical treatments [10]. Hence, the surgical management of grade III laryngeal collapse is controversial [9].

Subtotal epiglottectomy (STE) was first described in 2009 by Flanders and Thompson as a salvage option in two dogs affected by epiglottic retroversion and/or entrapment [11]. STE aims to remove the epiglottis across its widest base, for the relief of airway obstruction [12]. Arytenoidectomy performed by diode laser photoablation was described as a successful treatment for canine laryngeal paralysis; the procedure increased the width of the rima glottides, and it is considered a quick and safe technique [13].

We hypothesized that STE associated with the ablation of unilateral arytenoid cartilage may be performed to treat stage III laryngeal collapse with the aim of increasing the air passage through the larynx, decreasing negative intraglottic pressure, and reducing airway resistance. The aim of the present study is to evaluate the clinical outcome in dogs affected by stage III laryngeal collapse after subtotal epiglottectomy associated with the ablation of unilateral arytenoid cartilage tissue. 

## 2. Materials and Methods

The study protocol was set out in accordance with institutional guidelines for research on animals and was approved by the Ethics Committee of the University of Teramo; in this study, all of the dog owners were fully informed about the procedures, and written informed consent was obtained. All of the clinical procedures and the care of the animals adhered to the internal rules of the University of Teramo.

The inclusion criteria were dogs affected by stage III laryngeal collapse according to the classification developed by Leonard (the collapse of the corniculate processes of the arytenoid cartilages and the destructuration of the dorsal portion of the rima glottidis) and persistent severe dyspnea despite the initial surgical management of brachycephalic airway obstruction syndrome (BAOS) (staphylectomy, wedge rhinoplasty, and/or sacculectomy). The data were collected in the period between May 2018 to May 2021. 

For each dog, a complete signalment and history were noted. A general clinical examination was then performed, and any dog affected by clinically relevant cardiopulmonary, orthopedic or neurological morbidity was excluded from the study in order to avoid compromising the effort test.

After clinical examination, all of the dogs underwent an exercise test which consisted of 5 min of walking and 3 min of trotting. In accordance with the clinical respiratory score (CRS) elaborated by Riggs, the dogs were classified as follows: grade 0 refers to no abnormalities; grade 1 refers to mild or moderate stertor; grade 2 refers to moderate to severe stridor, moderate to severe inspiratory effort, and mild dyspnea; and grade 3 refers to severe stridor and stertor, and severe inspiratory effort, severe dyspnea, cyanosis or syncope may be present [14].

A blood gas analysis, complete blood count, and blood biochemistry test were performed. All of the dogs underwent a complete upper airway endoscopic examination using a fiberscope 60001VLK2 (Karl Storz, Verona, Italy). 

The dogs were sedated with medetomidine (Domitor®, Vetoquinol, Forlì, Italy) (10 μg/kg, IM) and methadone (Semfortan®, Dechra, Torino, Italy) (0.2 to 0.4 mg/kg, IM), and anesthesia was induced with propofol (PropoVet Multidose®, Zoetis, Roma, Italy) (4 mg/kg, IV). A radiographic examination of the thorax in two projections was performed in order to assess the lower airway and any concurrent problems, such as tracheal hypoplasia, aspiration pneumonia, pulmonary edema, and hiatal hernia, which are associated with BOAS. Subsequently, pharyngoscopy and laryngoscopy were performed to check the larynx and arytenoid functions and the appearance of the investigated structures in order to determine the stage of laryngeal collapse. A tracheoscopy by bronchial bifurcation evaluation was then carried out. The dogs undergoing surgery were intubated with a cuffed endotracheal tube, and the maintenance of anesthesia was achieved with sevoflurane (SevoFlo®, Ecuphar, Milano, Italy) in oxygen.

### 2.1. Surgical Technique

The dogs were placed in sternal recumbency, with the neck extended, the head fixed to the operating table both dorsally and ventrally, the mouth kept open, and the tongue pulled forward. A subtotal epiglottectomy and the ablation of the arytenoid cartilage were performed using an Airplasma® device (Onemythis, Torino, Italy) in continuous wave mode under constant video-assisted endoscopy.

The epiglottis was grasped with Allis forceps and a V-shaped mucosal incision was made by Airplasma®, which was extended from the left aryepiglottic fold to the epiglottic vallecula, then to the contralateral aryepiglottic fold. The incision was extended deep into the submucosal and muscular layers to expose the epiglottic insertion on the thyroid cartilage.

Following the epiglottectomy, the ablation of the cuneiform process and the inter-arytenoid band were carried out by the use of Airplasma® under constant endoscopic observation, as described by Olivieri [13]. During the procedure, the endotracheal tube was protected using a straight malleable retractor to prevent the contact of the tube with the instrument. Under constant endoscopic observation, the ablation of the arytenoid cartilage was performed; it consisted of the ablation of a portion of the corniculate process of the left arytenoid cartilage, followed by the removal of the ablated portion using a gauze sponge. The portion of the ablated cartilage was limited caudally by the laryngotracheal junction, and it was extended from the interarytenoid band to the cuneiform process of the arytenoid cartilage (Video S1).

Once the procedure was completed, the surgical site was monitored for two minutes in order to check that there was no hemorrhage, and to manage any possible intra-operative complications. A tracheostomy tube was prepared in the case of immediate postoperative laryngeal edema. 

The operative times were recorded, and the surgery time was counted from the first epiglottis incision to the end of the unilateral arytenoidectomy. 

### 2.2. Post-Operative Management

All dogs recovered in the intensive care unit (ICU) in the 24 h following surgery. The post-operative medication included methadone (0.2 mg/kg IM every 4–6 h), cefazolin (22 mg/kg IV every 12 h until the dog accepted oral antibiotics), dexamethasone 21 phosphate disodium (0.1 mg/kg SC, a single dose), and fluid therapy with Ringer’s lactate solution (4 mL/kg/h IV). The patients received continuous assistance, and particular attention was paid to the type of breath and respiratory rate. The dogs with stable respiration and without gastrointestinal signs were discharged from the ICU to standard wards. Stable patients were offered a meat meal and oral medications including amoxicillin-clavulanic acid (20 mg/kg PO every 12 h) and tramadol (2–4 mg/kg PO every 8 h) for up to 7 days after the surgery. The patients were discharged 48–96 h after surgery. 

Post-operative complications were classified as major in the event of surgical revision, i.e., mild/severe intraoral bleeding and severe local edema. Minor complications including local edema and slight self-limiting bleeding do not require revisional surgery.

In the case of major postoperative complications, a tracheostomy kit was prepared for each patient.

The patients were observed one month after surgery; a complete physical examination was performed in order to assess the respiratory effort test as previously described. 

When possible, one year after surgery, telephone interviews were conducted according to the model of Riecks et al. [15]. One of the authors asked the owners to rate their dog’s surgical outcome on a scale, as shown in the Table 1. 

## 3. Results

Sixteen dogs matched the inclusion criteria: ten French bulldogs, two Chihuahuas, one English bulldog and three mixed-breed dogs. In the study population, there were six entire males, seven entire females, and three neutered females. The median age of the dogs was 3.2 years (ranging from 2.5 to 5 years). The median bodyweight of the dogs was 7.3 kg (ranging from 3 to 15 kg) (Table 2). 

All of the dogs were referred with evidence of respiratory distress including inspiratory dyspnea, stridor, and stertor. Other clinical signs among the 16 dogs were exercise intolerance (three dogs) and cyanosis (two dogs). 

After the preoperative exercise test, the dogs were classified as grade 1 in one animal, grade 2 in six animals, and grade 3 in nine animals (Table 2).

The thoracic radiographs were considered within the normal limits in all dogs. No evidence of tracheal hypoplasia was documented. 

All of the dogs underwent pharyngoscopy, laryngoscopy, and tracheobronchoscopy. The endoscopic examinations showed stage III laryngeal collapse in all of the dogs. Laryngoscopy revealed that the collapse of the corniculate processes was present during inspiration, which accounted for the persistent stridor and dyspnea. 

The surgical procedures performed from 18 to 24 months before the epiglottectomy and the photoablation of the arytenoid cartilaginous tissue included staphylectomy in all of the dogs (16/16), with three of them having laryngeal sacculectomy (3/16) or rhinoplasty (14/16). 

**Figure 1:**
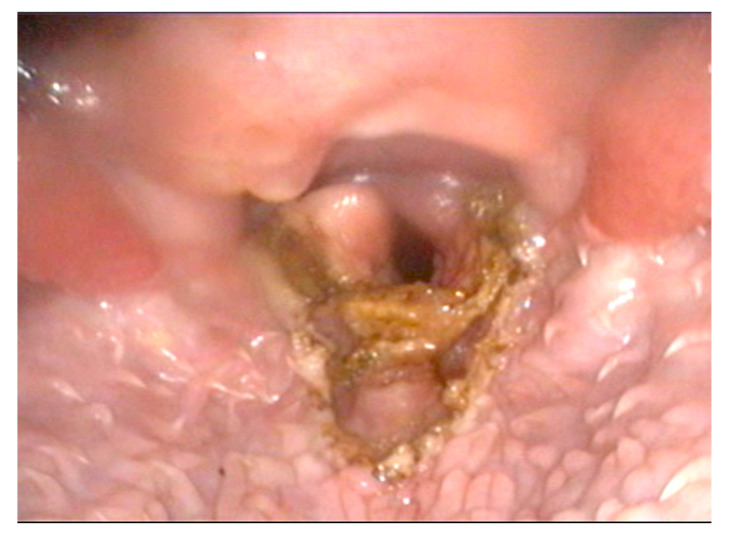
Larynx, immediate post-operative appearance: subtotal epiglottectomy and photoablation of the cuneiform process performed with the Airplasma device.

All of the patients underwent subtotal epiglottectomy and the photoablation of unilateral arytenoid cartilage (Figure 1). The surgical meantime was 31.3 min (range: 25 to 45 min).

After the subtotal epiglottectomy and arytenoid photoablation, a revision surgery was required. In two dogs (#3 and #7), a temporary tracheostomy was performed, as laryngeal edema was observed during surgery. Four days postoperatively, a laryngoscopic evaluation was performed in order to assess laryngeal functionality, and the tracheostomy tube was removed.

One month after the surgery, a complete physical examination was performed. The effort test was repeated, and the dogs were classified according to CRS as follows: grade 0 (eight animals), grade 1 (six animals), and grade 2 (two animals). 

One year after surgery, the owners of twelve patients rated the quality of life and outcome of the dogs as follows: excellent in five cases, good in five cases, and fair in two cases (Table 2). 

## 4. Discussion

Brachycephalic obstructive airway syndrome is characterized by clinical signs such as exercise intolerance, dyspnea, vomiting, regurgitation, and pathological respiratory noises including stertor and stridor. Episodes of severe dyspnea, when they occur, can lead to cyanosis, hyperthermia, and also syncope. The clinical signs are accentuated around one year of age and persist or worsen throughout life, sometimes even with medical and surgical management [16]. The aim of surgery is to improve inspiratory function by resecting the redundant portion of the soft palate, treating stenotic nostrils, and—in some cases—resecting everted laryngeal saccules [17]. Folded flap palatoplasty (FFP) and staphylectomy are the more frequently performed surgical procedures. FFP involves the removal of a part of the oropharyngeal mucosa in order to make the palate thinner, and the remaining caudal portion of the palate is retracted rostrally and sutured folded on itself. Staphylectomy involves the full-thickness removal of the exuberant portion of the palate [18]. Although all of the patients included in the study had a BAOS diagnosis and underwent one or more of these surgical procedures, they exhibited clinical symptoms and were diagnosed with grade III laryngeal collapse as a result of chronic upper airway obstruction 18-24 months after their first surgical correction.

Laryngeal collapse results in chronic upper airway obstruction, and is most often associated with BAOS [1]. The incidence of laryngeal collapse varies from 50% to as much as 95% in dogs affected by BAOS [2]. In 2009, De Lorenzi suggested that bronchial collapse is a common finding in brachycephalic breeds, and that laryngeal collapse is significantly correlated with more severe bronchial collapse, which could be the result of excessive respiratory efforts in young dogs with more supple cartilages [19]. The literature reports that the size of the rima glottidis is smaller in pug dogs; these breeds are also significantly more often affected by severe laryngeal collapse than French bulldogs [20]. In contrast to this information, the present study population included mostly French bulldogs (8/12 patients) and no pugs. Laryngeal collapse is also reported in small breed, non-brachycephalic dogs as a result of chronic airway obstruction and/or as a congenital condition [9]. In the present study, a population of non-brachycephalic dogs was included. All of the dogs were previously treated surgically with staphilectomy because they presented signs which were attributable to the obstruction of the upper airways.

The management of stage II and III laryngeal collapse is still controversial [8,21]. Most authors consider that dogs affected by these conditions carry a guarded prognosis. More recently, a study reported that dogs with moderate and severe laryngeal collapse improved their clinical condition after the surgical correction of the abnormalities associated with brachycephalic syndrome [1]. 

In 2012, White reported the outcome of the 10 out of 12 dogs affected by stage III collapse; this was confirmed when the left-sided arytenoid laryngoplasty was performed. The surgical procedure allowed them to obtain an enlargement of the rima glottidis and a significant improvement of the clinical conditions. The patients’ owners, about 3.5 years after surgery, reported significant improvements in exercise tolerance, respiratory function and, therefore, quality of life [8].

The ideal surgical procedure to manage laryngeal collapse would be minimally invasive, low in cost, last the lifetime of the patient, and result in a return to the normal activity of the patient [21]. Unfortunately, none of those surgical procedures are without risk and complications [21]. 

Arytenoid laryngoplasty requires a surgical approach in the neck region, in proximity to the laryngeal nerve, the jugular vein and the esophagus [22]. Although it could be an effective technique, it is associated with several complications, including aspiration pneumonia, seroma, intramural hematoma, postoperative dyspnea or edema, surgical failure from suture breakage or arytenoid cartilage fragmentation, and the persistence of respiratory signs [23]. In the study that was previously mentioned, seven dogs were subjected to arytenoid laryngoplasty to manage laryngeal collapse and required temporary tracheostomy, and two dogs were euthanized during the follow-up period due to the ingravescence of their clinical respiratory signs [8]. Furthermore, the procedures used in the present study seem to have results which are subjectively comparable to the previous one in terms of the enlargement of the rima glottidis with short surgical times and less of a complication rate; only two French bulldogs were subjected to tracheostomy. Temporary tracheostomy is a common complication after laryngeal surgery, especially in brachycephalic dogs [24]. For these reasons, the surgeon and the ICU staff should be prepared to manage this complication. In the present study, major intraoperative complications were observed. In two French bulldogs, temporary tracheostomies were performed in order to manage the upper airway respiratory edema and prevent other postoperative complications; in these dogs, the laryngeal cavity was subjectively very narrow. In both patients, the tracheostomy tube was removed in four days, and they were discharged two days later. One year after surgery, both dogs had a fair clinical outcome according to the telephone interviews. In the present study, the tracheostomy was associated with worse outcomes compared to the other patients. Despite this, it was not possible to conduct a statistical survey due to the small number of patients included in the study. 

Permanent tracheostomy was the traditional treatment in dogs affected by grade III laryngeal collapse [23,25]. A paper published in 2018 reported that in 15 dogs who underwent permanent tracheostomy, five patients had a long-term good quality of life > 5 years [10]. This palliative treatment, however, is not appreciated by the owners, and it could be associated with some relevant complications such as aspiration pneumonia and the need for the revision of the tracheostomy due to skin-fold obstruction [26].

In the present study, all of the dogs were previously treated with staphylectomy to correct the excessive length of the soft palate. Although the surgical procedure was properly performed, evidence of exercise intolerance was reported by the owner and confirmed by the clinician. Mild or severe respiratory signs were also present, and respiratory effort was quantified after the clinical respiratory score (CRS) as previously described. 

Subtotal epiglottectomy (STE) is a surgical procedure of the intraoral natural orifice. Its aim is to remove the glottis in order to improve the amount of air passing through the laryngeal cavity [12]. STE was previously performed as a surgical treatment in dogs affected by epiglottic reversion. In a study population of 50 dogs, intraoperative and major postoperative complications and the survival of the dogs undergoing the surgical management of epiglottic retroversion were reported. Thirteen dogs were treated by STE. The authors reported one intraoperative complication (difficulty closing mucosa in overexposed epiglottic cartilage), one short-term major complication (airway obstruction after extubation, which was resolved with rostral tongue traction until the dog was awake), and one long-term major complication (the development of severe seizures, aspiration pneumonia, and death 6 months postoperatively). STE was associated with a lower complication rate (16.7%) in comparison with non-incisional epiglottopexy (53.5%), incisional epiglottopexy (50%), partial epiglottectomy (66.7%), and other surgical procedures [27]. STE was also reported as a successful surgical treatment in a dog affected by epiglottis chondrosarcoma [28]. The main function of the epiglottis is to protect the lower airway against the aspiration of liquids and solids during swallowing [28]. Several studies published in human and veterinary medicine reported that the epiglottis is not essential during swallowing, as some protection is also provided by the closure of the glottis, the extreme sensitivity of the laryngeal mucosa, the cessation of respiration during deglutition, and the protection offered by the base of the tongue [28,29]. In the case of epiglottectomy, aspiration pneumonia could not be excluded. An experimental study evaluated the airway protection during swallowing guaranteed by different anatomical structures; the authors claimed that the epiglottectomy had no effect on any phase of aspiration, which suggests that, although the epiglottis may assist in airway protection, it may have no essential role in this function [30]. On the other hand, subjectively, the epiglottis may decrease the airway flow in a patient affected by stage III laryngeal collapse. Thus, the authors may speculate that STE decreased the intraglottic luminal pressure, increasing the airway flow.

In addition to STE, the authors performed an ablation of the corniculate process cartilage in order to further enlarge the laryngeal area [1,31]. Video-assisted partial arytenoidectomy by diode laser photoablation was described as a successful treatment for canine laryngeal paralysis; it was described as a relatively quick and safe to increase the width of the rima glottidis [13]. Based on the authors' results, the technique described can be considered a valid strategy for grade III laryngeal collapse treatment.

In our study, after the effort test—as described in a previous paper—the grading was based on the clinical evaluation of the patients [14]. Whole-body barometric plethysmography would be a modality to objectify upper airway obstruction in brachycephalic dogs affected by laryngeal collapse [32].

According to telephone interviews carried out one year after surgery, the owners reported that physical exercise was better tolerated than was observed before the surgical correction. No dogs developed signs of aspiration pneumonia. Mullis reported one case of aspiration pneumonia after subtotal epiglottectomy in a dog affected by onset seizures which may have led to aspiration pneumonia. The authors were not able to exclude that the seizures were related to the surgical procedure [27].

STE and arytenoid ablation were performed using the Airplasma® device. The Airplasma® technology transforms air into an ideal conductor of plasma energy. The energy flow applied at 50 °C allows us to obtain tissue vaporization at the same time, with minimal thermal damage, and capillary hemostasis [18,33]. The Airplasma® device was first applied in 2019 in dogs with oversized soft palates who underwent palatoplasty. In two dogs, the authors reported mild bleeding after the surgery. 

The main limitation of the study was the subjective assessment of respiration by the owners one year after surgery. It would be advisable to evaluate the plethysmography in order to obtain an objective evaluation of the respiratory function and compare the pre- and postoperative data. It would also be interesting to obtain a long-term follow-up of the patients involved. Another main limitation is the small number of patients under study. To the authors' knowledge, stage III laryngeal collapse is an infrequent condition and few studies with a small number of cases similar to the present study have been published; therefore, further evaluations are strongly encouraged. 

## 5. Conclusions

In conclusion, this pilot study suggests that epiglottectomy associated with the ablation of the unilateral arytenoid cartilage improved clinical symptoms in dogs, and thus may be considered a valid surgical procedure to treat dogs affected by grade III laryngeal collapse. The patient follow-up provided encouraging results, and the dogs’ owners rated the outcome of the dogs undergoing surgery as satisfactory. 

## Figures and Tables

**Table 1:** Clinical findings and respective scores of the dogs at the time of follow-up.

Clinical Signs	Score
Marked improvement in clinical signs with no restriction on physical activity	excellent
Improvement in clinical signs with some limits on physical activity	good
No improvement in clinical signs	fair
Severity of clinical signs increased after surgery	poor

**Table 2:** Pre- and post-surgery data of the 16 dogs included in the study.

Dog	Breed	Sex	Weight [kg]	Age (Years)	Clinical Signs before Surgery	Previous Surgical History	Clinical Respiratory Score (CRS) before Surgery	Surgical Time [Minutes]	Complications	Clinical Respiratory Score (CRS) One-Month Post-Surgery	Clinical Signs One-Month Post-Surgery	Telephonic Follow up
1	French bulldog	F	6.2	2.5	inspiratory stertor/stridor	palatoplasty, rhinoplasty	3	30	-	1	clinically well, no signs of regurgitation or swallowing dysfunction	excellent
2	French bulldog	M	8.4	3	inspiratory stertor/stridor	palatoplasty, rhinoplasty	1	20	-	0	clinically well, respiratory function improved and stable, no signs of swallowing dysfunction	excellent
3	French bulldog	F	7	3.5	inspiratory stertor/stridor	palatoplasty, rhinoplasty, sacculectomy	3	40	Postoperative edema, temporary tracheostomy	2	signs of baos, panting, intermittent regurgitation	fair
4	French bulldog	F	7.6	2.5	cyanosis, exercise intolerance	palatoplasty, rhinoplasty	3	35	-	1	no signs of baos, respiratory function improved and stable	good
5	French bulldog	FN	8	4	inspiratory stertor/stridor	palatoplasty, rhinoplasty	2	25	-	0	no clinical sign of baos, respiratory function stable.	excellent
6	French bulldog	M	10.3	3.5	inspiratory stertor/stridor, exercise intolerance	palatoplasty, rhinoplasty	2	40	-	1	clinically well, respiratory function improved and stable	good
7	French bulldog	F	9	4	inspiratory stertor/stridor	palatoplasty, rhinoplasty	3	28	Postoperative edema, temporary tracheostomy	2	stertor storing and excessive panting, regurgitation	fair
8	French bulldog	F	7.5	3	inspiratory stertor/stridor	palatoplasty, rhinoplasty	3	37	-	0	no signs of baos, respiratory function improved and stable	excellent
9	Chihuahua	FN	3	3	inspiratory stertor/stridor, cyanosis	palatoplasty.	3	30	-	1	clinically well, respiratory function improved and stable	good
10	Chihuahua	F	2.5	4	inspiratory stertor/stridor	palatoplasty.	2	45	-	0	no clinical sign of baos, respiratory function stable.	good
11	Mixed breed	M	5	5	inspiratory stertor/stridor	palatoplasty, rhinoplasty	2	28	-	0	no clinical sign of baos, respiratory function stable.	excellent
12	Mixed breed	M	9	4	inspiratory stertor/stridor	palatoplasty, rhinoplasty sacculectomy	3	30	-	0	no clinical sign of baos, respiratory function stable.	good
13	French bulldog	FN	7	4.5	inspiratory stertor/stridor	palatoplasty, rhinoplasty	3	26	-	0	no clinical sign of baos, respiratory function stable	-
14	French bulldog	F	5.5	3	inspiratory stertor/stridor, exercise intolerance	palatoplasty, rhinoplasty	2	28	-	1	clinically well, respiratory function improved and stable	-
15	English bulldog	M	15	2	inspiratory stertor/stridor	palatoplasty, rhinoplasty	3	27	-	1	clinically well, respiratory function improved and stable	-
16	Mixed breed	M	7	3	inspiratory stertor/stridor	palatoplasty, rhinoplasty	2	31	-	0	no clinical sign of baos, respiratory function stable	-

## Data Availability

Data are contained within the article or Supplementary material.

## Supplementary Materials

The following supporting information can be downloaded at: https://www.mdpi.com/article/10.3390/ani12091118/s1, Video S1: Surgery procedure.
